# Hospital Contracts and Prices

**Published:** 1910-05-21

**Authors:** 


### SPECIAL DEPARTMENTAL ARTICLE.

Contributions and questions on matters affecting this department of an institution are invited.

### HOSPITAL CONTRACTS AND PRICES.

### VI.—DOMESTIC.

#### RENEWAL OF FURNITURE.

IT is almost impossible to arrange a contract covering a period for articles purchased under the sub-head "Renewal of Furniture." As a rule the requirements are somewhat isolated, consisting usually of new chairs, new carpets, upholstering existing furniture, renewing ward furniture, and repairing articles broken. It is practically impossible to foresee what will be required, and consequently a fixed contract for a year or two or three years is out of the question. It is best to deal with the requirements as they arise and obtain a definite price before passing an order. If there is a works department, a great many articles can be manufactured without difficulty in the institution's own workshops; but as a rule, if a number of the same article is to be ordered, it is cheaper to obtain estimates from good firms with ample machinery than to manufacture in the institution, unless the latter's workshops are equipped with more machinery than is usually the case. If prices are required from several firms, it is an excellent plan to prepare drawings of the articles required and a definite specification describing the method of manufacture, so that all may tender on similar lines, otherwise it will be found that each firm is tendering for a different article, the competition is unfair, and if the lowest price is accepted it frequently means that the worst article is purchased.

It is of great assistance if, before inviting tenders, one of each of the articles required is made in the institution's workshops and exhibited to the tendering firms as the standard article to which they must work. With regard to these patterns, care should be taken that the lines of the furniture required for ward use are as clean cut as possible. There should be no angles for the lodgment of dust; legs of tables should be turned smooth without the grooves which are so popular in most articles of domestic furniture. A perfectly even curve in a leg of a table or chair will add the character which is required to relieve the severity of an absolutely straight leg. If panels are allowed at all in any locker or cupboard, they should have rounded corners, but a solid door is preferable. Dressing trolleys, wash-hand trolleys, etc., should be made of glass and enamelled iron, but for lockers it is probably preferable to have hard wood, well varnished, with marble or glass tops. The glass and enamelled-iron locker sometimes seen in hospitals, besides being more costly soon becomes unsightly, as, unless porcelain enamel be used, the surface chips very readily and the smartness of the ward is destroyed.

If firms are to be invited to compete for numerous articles of cabinet-makers' work, a definite form of contract is advisable, and that which is here given can be recommended as having been found useful.

NAME OF INSTITUTION.

This Tender, issued to......................., of......................, is to be delivered to the Board of Management, .............. at 9 A.M. on....................., marked on the envelope "Tender for Cabinet Maker's Work, etc.," without which the Tender may not be considered.

......................., Secretary.

*Cabinet Maker's Work at.....................*

......... hereby Tender to supply at the above Institution the Articles as per Schedule, in accordance with and agreeably to the Conditions and Schedule attached.

Dated this.........day of.........................., 1908
Signature ..........................................
Address ..........................................
Witness ..........................................
Address ..........................................

Conditions of Contract and Specification for the Manufacture and Supply of Cabinet Maker's Work for the Board of Management of .....................

Date, .......................

*Conditions of Contract.*

(1) To be in accordance with specification. — The several articles of furniture shall be made strictly in accordance with the patterns, drawings, and specification, and to the entire satisfaction of the Board of Management, their Secretary, or such other person as they or he may appoint.

(2) Reservation as to work to be allocated to Contractor. — Contractors may tender for the whole or any part of the furniture included in the Schedule, but the Board reserve the right to determine the amount of work to be done by individual contractors and to apportion the same as they may deem expedient.

(3) To be manufactured on Contractor's premises and right of access. — All furniture, except (any exception, such as Windsor chairs, etc.) shall be made on premises occupied by the Contractor, and any member of the Board, their Secretary, or other representative appointed by the Board, shall at all reasonable times have access to the workshops for the purpose of examining the goods in the course of manufacture.

(4) Quality and right of rejection. — All furniture, locks, fittings, and materials of every description shall be of the best quality, and the Board, their Secretary, or such other person as they or he may appoint, shall have full power to reject any goods or thing which in their or his opinion are not in accordance with the patterns, drawings, and specifications, or unsatisfactory in quality.

(5) Default. — Should the Contractor refuse to replace or rectify, within a reasonable time after written notice has been sent to him, any goods rejected or deemed unsatisfactory, the Board shall be entitled to obtain such goods elsewhere, and the cost thereof, with all incidental expenses arising in connection, shall be borne by the Contractor, and any money due, or becoming due, to the Contractor under this Contract may be retained by the Board till such costs have been discharged.

(6) Sub-letting, etc. — The Contractor shall not transfer, assign, or underlet, directly or indirectly, his contract, or any part, share, or interest therein without the permission of the Board to be given in writing.

(7) Price to include delivery and depositing. — The price to be paid for the respective goods shall include for the delivery of same, and for the depositing of the same in such places as may be directed.

(8) Delivery and penalty. — All goods shall be delivered between ................., and should the Contractor fail to complete his contract by the latter date, he shall pay to the Board by way of liquidated damages a sum equal in amount to 5 per cent. of the amount of his contract for each and every week he may be in default.

(9) Method of payment. Eighty per cent. of the amount of the Contract will be paid on satisfactory completion of the Contract, and the remaining 20 per cent. within three calendar months of the date of such completion, subject to the following clause.

(10) Faults and defects to be rectified. Should any faults develop, or defects in workmanship or materials be discovered during the three months above mentioned, the Contractor shall at his own cost immediately rectify the same, and any moneys due to him will be withheld until the Board's Secretary certifies that all goods are satisfactory.

(11) Bankruptcy. In case the Contractor should become bankrupt or insolvent, or compound with his creditors, or commit any act of bankruptcy, all goods which have been previously delivered and accepted as satisfactory shall be considered to be the property of the Board, who will pay for same at rates proportionate to the Contract prices, and in such case the Board shall be at liberty (on notice being given to that effect by the Board's Secretary) to determine the Contract so far as regards the remainder of the goods comprised in the Contract.

(12) Saving clause *re* acceptance. The Board do not bind themselves to accept the lowest or any Tender.

Signed........................

*Specification of Cabinet-maker's Work.*

The whole of the furniture must be constructed with the best workmanship and materials in accordance with the drawings and such further details as may be provided.

The wood to be the best of its kind, free from sap, large or dead knots, stains or other defects, and to be thoroughly seasoned.

All figured sizes and thicknesses to be net when finished.

All mouldings to be worked on the solid as shown on detail drawings.

The sides, backs, and bottoms of drawers, unless otherwise shown, are to be in canary wood, dovetailed at back and front, and all drawers are to have hard wood runners.

Where joints are required to obtain the necessary width, they must be well and truly made, and either dowelled or cross-tongued; if the latter, the tongue must be stopped short at all fair ends.

All table-tops must be buttoned to frames with either hard wood or iron buttons, and where the length of table exceeds 5 ft. they are to have intermediate cross rails for that purpose.

All furniture for Sitting or Bed rooms to be French polished in the best manner on all exposed faces, the backs and insides of wardrobes, etc., to be slightly stained and half polished. All ward furniture to have two coats of best copal varnish inside and out.

Articles shown in drawings to be tiled are to be finished with the best quality plain glazed tiles of approved colour, and are to be securely fixed in cement.

The mirrors to be of the best quality, free from specks or other defects. All edges of mirrors to be blacked.

Provide all necessary ironmongery of approved quality, whether the same be specially mentioned or not.

The upholstered chairs and couches to be stuffed with best black hair, samples of which must be submitted for approval.

The marble to be St. Ann's, or other of equal quality approved by the Board, of the thicknesses shown on drawings, well polished on all exposed faces.

